# Diagnostic disclosure in Alzheimer’s Disease: A review

**DOI:** 10.1590/S1980-57642009DN20400006

**Published:** 2008

**Authors:** Irina Raicher, Paulo Caramelli

**Affiliations:** 1 Division of Neurology of the Hospital das Clínicas, University of São Paulo School of Medicine, São Paulo, Brazil.; 2 Behavioral and Cognitive Neurology Research Group, Department of Internal Medicine, Faculty of Medicine, Federal University of Minas Gerais, Belo Horizonte, Brazil.

**Keywords:** dementia, Alzheimer’s disease, truth disclosure, diagnosis disclosure, bioethics

## Abstract

Although growing, the literature on research into attitudes of general and specialized physicians towards disclosing the diagnosis of dementia and Alzheimer’s disease (AD), or the current practice on AD disclosure, remains limited. Moreover, information is also scarce on what caregivers, or indeed patients themselves, wish to know with regard to their diagnosis. The goal of the present article was to present a review of the current available literature on the topic of truth telling in dementia, especially in AD. The studies discussed in this review were mainly conducted in Europe, particularly in the United Kingdom, as well as the United States. Disclosure of AD diagnosis is not a common practice among physicians. In the clinical context, the discussion on diagnosis disclosure can be valuable for improving the care of AD patients and their families.

## Abstract (pt)

O conhecimento científico ainda é limitado, embora crescente, a respeito das atitudes de médicos generalistas e especialistas sobre a revelação do diagnóstico de demência e da doença de Alzheimer (DA), ou ainda em relação à prática atual na revelação diagnóstica da DA. As informações também são escassas sobre o que cuidadores e os próprios pacientes querem saber sobre o seu diagnóstico. O objetivo deste artigo é apresentar uma revisão da literatura disponível atualmente sobre revelação diagnóstica em demência, especialmente na DA. Os estudos discutidos nesta revisão foram feitos principalmente na Europa, a maioria no Reino Unido, e nos Estados Unidos. A revelação diagnóstica da DA não é uma prática comum entre os médicos. No contexto clinico, esta discussão pode ser útil para melhorar o tratamento dos pacientes com DA e a atenção a seus familiares.

A significant shift in attitudes towards disclosure of the diagnosis of serious medical conditions has become evident in the past decades, from medical paternalism to one considering patient autonomy. Most of the research on truth-telling in relation to diagnosis has been in the cancer literature where this issue has evolved to the point that disclosure of cancer diagnosis has become the ethical norm in medical practice.

Nonetheless, although growing, there has been little interest in the research area concerning attitudes held among physicians towards disclosing the diagnosis of dementia, particularly Alzheimer’s disease (AD), or about what is the current practice of doctors in this field. Moreover, information is also scarce on what caregivers, or indeed patients themselves, wish to know with regard to their diagnosis. This issue is of great relevance in the light of the current treatment options for dementia and, even more importantly, in view of the introduction of potential new therapies for AD on the horizon.

The advantages and disadvantages of disclosing dementia diagnoses and the ethical issues involved are the subject of lively debate. The American Medical Association (AMA) guidelines for the diagnosis and treatment of dementia state that the diagnosis should be given directly to the patient “if at all possible”.^1^ However, it has been suggested that these guidelines are inadequate to address the clinical complexities of this issue and also the cultural diversities of patients and countries. In this sense, studies which address diagnostic disclosure of dementia in developing countries are still lacking.

The goal of the present article was to present a brief review of the current available literature on the topic of truth telling in dementia, especially in AD.

## Clinical and epidemiological aspects of dementia and AD

The world population, especially in developing countries like Brazil, is experiencing an accelerated aging process that has resulted in an increasing prevalence of chronic illnesses, such as Alzheimer’s disease (AD).^2,3^ Recent projections show that the proportion of people with dementia in developing countries is set to rise significantly over the next three decades.^4^ AD onset commonly occurs at the age of 65 years onwards and entails a significant shortening of the patient’s lifespan,^5,6^ usually of a progressive nature, with a mean of eight years from the beginning of symptoms to death and sufficient to cause significant functional impairment.^7^ Therefore, the ethical issues concerning this disease, which impairs not only patient quality of life but also of those around them, have come to the fore.^8-11^

The overall assistance of those with dementia is not only a challenge to physicians’ professional skills but also confronts us with difficult questions about the limits of individual autonomy and medical paternalism, the dignity of individuals, and their best interests.

For example, there is little research on the impact of disclosing a diagnosis of dementia, or how to decide whether a dementia patient is able to understand a diagnosis, or how much information to impart.

## Diagnostic disclosure in AD

### Practitioners’ attitudes

A study conducted in Nottingham, United Kingdom, investigated current practice and attitudes on diagnostic disclosure in dementia among geriatricians and old-age psychiatrists.^12^ The authors found that only 40% of these specialists regularly tell patients the diagnosis and 20% see no benefit in such disclosure. However, 72.5% of the respondents would wish to know if they were diagnosed to be suffering from the illness. Although physicians were aware of many benefits of disclosure, they had concerns regarding the certainty of diagnosis, the patient’s insight, and potential detrimental effects like causing distress and destroying hope or motivation. Other studies carried out in the United Kingdom, examining the practice of general practitioners, geriatricians and psychiatrists have shown similar findings^13-17^ Rice et al., who surveyed geriatricians and psychiatrists, found a relationship between diagnosis disclosure and dementia severity: patients with mild and moderate forms were told their diagnosis more commonly than patients with severe dementia.^15^ The wide variation in practice suggests that further debate was needed on this issue.

Another study in France examined whether and how diagnosis of AD is disclosed by French general practitioners. Only 28% of them reported having disclosed diagnosis to the patient and few discussed the consequences of AD and symptoms with patients, whereas they have a less-reluctant attitude toward caregivers.^18^

We have recently conducted a study in Brazil, in which a questionnaire devised to survey AD diagnosis disclosure was sent to geriatricians, neurologists and psychiatrists who regularly assist dementia patients. Only 44.8% of the 181 physicians who responded stated that they regularly disclose the diagnosis to patients, although 85.6% of them use clear terminology. Despite their usual practice, 76.9% of these physicians would want to know their diagnosis if affected by AD.^19^

### Caregiver attitudes

Several studies have investigated the views and opinions of family caregivers about diagnostic disclosure. Maguire et al. interviewed 100 family members accompanying patients with AD in Ireland: 83% of them expressed a wish that their relative should not be told and the main reason for withholding information was preventing depressive illness.^20^ Despite this, 71% of the same caregivers indicated that they would like to be told if they were developing AD. Moreover, 75% said they would like to be submitted to a predictive test for AD, if such a test was available, since this information “would help them make provisions for their future and thereby reduce the burden on their families”.^20^

A smaller study reported by Barnes in 1997 provides some contrasting evidence.^21^ In this study, 17 out of 30 first-degree relatives wanted their afflicted family member to be told. Among reasons offered were the following: “It’s no use hiding it”, “They could try to keep their mind working”, “They could sort out their legal affairs” and “They could explain why they couldn’t remember things”.^21^

Another study by Holroyd et al., in the United States, examined the relationship between opinions on AD disclosure and knowledge about the disease.^22^ In this study, nearly half of the 57 family members of AD patients from community associations for relatives/patients with dementia, expressed that they had not received enough information about dementia. Most family members considered that patients should be disclosed their diagnosis and prognosis by the physician, although half reported that the patient had a bad reaction to disclosure and only 1/3 considered the attitude of disclosing helpful for the patient.

In 2003, Pucci et al. interviewed the closest relatives of 71 Italian subjects diagnosed for the first time as having AD, using a semi-structured questionnaire.^23^ Spontaneous requests by relatives to not communicate issues concerning the diagnosis were also recorded. Forty-three (60.6%) relatives spontaneously requested that patients should not be fully informed. After being interviewed, nobody thought that the patient should be given all the information. Justifications were related to the fear of the onset or worsening of depressive symptoms in the patient, a similar result found by Maguire et al. (1996), indicating that the family caregivers worry about the emotional impact of disclosure to the patient.

In Taiwan, Lin et al. found that an overwhelming majority (93%) of subjects favored disclosure of the diagnosis if, hypothetically, they personally were affected by AD.^24^ However, a smaller majority of family members (76%) favored disclosure of the diagnosis to current AD patients.

In a more recent study conducted in Brazil, we found that disclosure of AD diagnosis was approved by 42.5% of 40 family caregivers and that this rate was not influenced by socioeconomic level.^25^

### Patient attitudes

A number of studies have sought the views of patients about diagnosis disclosure. Most of these studies interviewed patients prior to AD diagnosis.

A study in 1988 by Erde et al. involving 224 adults (less than 19% of whom were aged 65 years or older) showed that more than 90% wanted to be told of a diagnosis of AD so they could make plans for care, obtain a second opinion, and settle family matters.^26^

Holroyd et al. questioned 156 people living in a retirement community in the United States.^27^ Respondents (mean age of 79.7 years) were given vignettes of two patients, one with terminal cancer and one with AD. The key finding was that 79.5% of those questioned said they would like to know if they had AD disease, while 91.7% said they wanted to know if they had cancer. Besides, 65.7% would want their spouses to be told of an AD diagnosis, while 80.2% would want their spouses to be told about a possible cancer diagnosis. Reasons offered were similar in both cases: to allow advance planning, to “settle” family matters, to travel or take a vacation and to consider suicide while still able (<3.5% of responses). The authors concluded that these results “may support disclosure for most patients with AD, but clinical and ethical issues remain in individual cases”.^27^

Another study from Holroyd et al. expands on these previous data, revealing that patients aged 65 years or older and having personal experience with AD were significantly less likely to want to know themselves if they had AD than those without personal experience.^29^

Jha et al. interviewed 53 patients with dementia and 47 patients with depression using a postal questionnaire in the United Kingdom.^28^ Most depressed and dementia patients (>75%) liked the idea of knowing their diagnosis. Within the dementia group, the majority of patients with mild or severe dementia welcomed the idea of knowing their diagnosis and 13 (100%) of the patients with vascular dementia wished to know (compared with 68% cases with AD). However, among dementia patients who also happened to be depressed, a higher proportion (60%) expressed an unfavorable view towards knowing their diagnosis while a minority (40%) was actually upset. Older married females, especially those with depression and AD, felt pessimistic afterwards. There was no significant difference between patients with dementia or depression in their wish to know their diagnosis.^28^

O’Keeffe et al. asked consecutive patients admitted to a respiratory and a geriatric unit in Ireland whether and how they would wish to be told of cancer or AD.^30^ Of the 207 patients interviewed, 174 (84%) wanted to be told about cancer or dementia where the proportion who would wish to be told did not differ between older patients (89 out of 108 patients; 82%) and younger patients (85 out of 99 patients; 86%). Thirty patients (15%) sought reassurance during or after the interview, and 13 patients (6%) reported that they had been bothered by the questions. Of the 207 patients, cancer or dementia was diagnosed in 23 patients (11%). Preferences for disclosure or nondisclosure were honored for 20 patients (87%).

In a more recent study held in the United Kingdom, the views of patients prior to diagnosis were analyzed.^31^ Participants were a consecutive sample of patients aged 65 and over with memory complaints. They were asked what they considered to be causing their memory problems and whether or not they would want to know the actual cause. They were then specifically asked if they would want to know if diagnosed with AD and what their reasons were for this. Two-thirds of patients were uncertain regarding the cause of their memory difficulties although the remainder did offer some valid explanations. Eighty-six per cent wanted to know the cause while 69% wanted to know if diagnosed with AD. A variety of reasons were offered to support their decision.^31^

## Discussion

There are interesting data suggesting that some patients only wish to know the overall underlying cause of their memory problems and not to know they are diagnosed with AD. These findings show that any conversation concerning disclosure must be conducted with appropriate sensitivity since most of these individuals had not considered AD as a possible cause of their problem.^31^

Furthermore, studies about patient opinion present high percentages favoring disclosure compared to the current rate of disclosure practiced by physicians. This confirms that some unjustified attitudes are held by medical doctors and emphasizes the importance of making individual decisions.

Much more research is necessary investigating these issues, especially multicentre questionnaires to seek the views of patients, by asking whether or not they desire further information regarding prognosis, treatment options, and other aspects related to their diagnosis, and whether or not they would want their family members to be informed. Future research on the manner in which to disclose the diagnosis would help practitioners who often feel ill-prepared to deal with this task.^14^

Studies on other chronic diseases show that the interpersonal ability and the professional skills of the physician in the process of disclosing bad news profoundly affect the level of anxiety and hope experienced by patients and families, their adaptation to the disease and the promotion of effective relationships with health professionals.^32-34^

Empirical studies of diagnostic disclosure in dementia have largely focused on quantifying attitudes and practice, with less emphasis on the process and impacts of disclosure. The perspectives of people with dementia also remain greatly under-researched. Further qualitative research is needed to deepen our understanding of the process of disclosure and enable people with dementia and their carers to obtain as much, or as little, information about their diagnosis as they want.

## Conclusions

A wide range of opinions regarding diagnostic disclosure in AD was found by investigators in different countries, where between 68–90% of patients, 17–76% of carers, and 28–44.8% of clinicians are in favor of disclosure.

In studies with practitioners, certainty of diagnosis, severity of dementia, the patient’s insight, patient’s wish to be told, relatives’ views and potential detrimental effects causing distress and destroying hope or motivation were the main reasons that influenced disclosure. An inconsistent approach was revealed in that although a high proportion of doctors would like to know their diagnosis if affected, this belief was not reflected in their usual practice of disclosing to patients.

From the caregivers’ viewpoint, emotional impact was the main factor influencing the decision whether to tell patient the diagnosis. There is some inconsistency too, the studies showed that a higher percentage of caregivers expect to be told their diagnosis if they were affected with AD, than they expect their relatives to be told.

Finally, in patients’ (or potential patients’) opinion, the same inconsistency was found since when asked about disclosure of AD a high percentage favored disclosure for themselves, but were against informing their relatives.

Assessments of patients' and carers' needs were studied mostly in Europe, especially in the UK, as well as the USA. It would be valuable to expand scientific knowledge on outcome of disclosure in different cultures, including developing countries and non-occidental cultures.
